# External Manipulations of Fœtus in Utero to Rectify Supposed Malpositions—Vindication of the Action of the Scott County (Iowa) Medical Society in Expelling a Member for Alleged Unprofessional Conduct

**Published:** 1860-03

**Authors:** 


*External Manipulations of Fœtus in Utero to rectify supposed Malpositions—Vindication of the Action of the Scott County (Iowa) Medical Society in expelling a member for alleged unprofessional conduct.*—(Official.)

The "New York Medical Press," of January 28th, contains an editorial notice of an article, published in the same number, from Dr. I. Langer, of Iowa, in which editorial the following language is used :

"This, we think, is a thorough refutation of the charges brought against Dr. Langer by his late associates of the Scott County Medical Society, placing them in an unenviable light before the professional world."

It must appear a little remarkable to the profession that this expression of opinion should be made by the conductors of a medical journal, on the *ex parte* statement of a man who stands convicted of conduct unbecoming a physician and a gentleman, and of wilful violations of the code of ethics. It is no uncommon circumstance for a party arraigned for crime in courts of justice to make out a plausible story of persecuted innocence, and sometimes so successfully as to enlist the sympathy of all who may listen, until the evidence of guilt is presented. For a judge or jury to pronounce the verdict of not guilty, on the representations alone, of the party accused, would be even less unreasonable than the course pursued by the editors of the New York Medical Press. Less, because in this instance the party has been tried by a body of his peers and found guilty, and condemned as unworthy of their confidence and fellowship. The haste with which they have presumed to reflect unwarrantable accusations against this body of medical men, must rather place themselves, as editors, "in an unenviable position before the medical world." The members of the Scott County Medical Society fear not the verdict of the profession at large, relating to the course they have taken ; and, when all the facts are fully known, they are confident of being sustained by all who are hostile to quackery, and have the good of the profession at heart ; and, for the honor of the profession, they regret the hasty and inconsiderate step which has been taken by the editors of the above mentioned journal.

In due time, the action of this Association will, undoubtedly, be reviewed by the State Medical Society of Iowa, before which will be placed all the evidence in the case. But, in the mean time, it cannot permit such gross misrepresentations and unfounded opinions to pass unnoticed before the medical world.

The grounds on which the Society acted, and the position it has taken in relation to a certain kind of practice, are expressed in the following preamble and resolution passed at the quarterly meeting of the Scott County Medical Society, October 25th, 1859:

" *Whereas*, at the previous meeting of the Scott County Medical Society, held July 26th, 1858, Ignatius Langer was found guilty of a charge then preferred against him, of making and repeating from day to day certain unwarrantable examinations and manipulations of a pregnant female, previous to the time of labor, with the pretended object of discovering and correcting a malposition of the fœtus in utero, and of publicly proclaiming the object and intention of his repeated visits to said patient; And, whereas, said Langer, in the face of an unanimous vote of this Society condemning the practice, still persists in his avowed determination of requiring females to submit to any examination of their persons which he may think proper to make at any time during their pregnancy, which is contrary to all authority and usage, and derogatory to the dignity and decency which should ever characterize the conduct of a physician and gentleman; And, whereas, certain other charges, then preferred against him, which were submitted to the investigation of a special committee, have been well and fully substantiated by testimony adduced by various persons, members of this Society, and others, and which charges constitute special and distinct violations of the letter and spirit of the Code of Ethics, by which this Society is governed; And, whereas, during this investigation, said Langer has publicly uttered various contumelious remarks regarding the members of this Society individually and as an association of professional men, thus exhibiting his disregard of the opinions and actions of the Society, endeavoring to cast upon it the imputation of ignorance and the want of a generous spirit of tolerence; and whereas, this Society deems it due to its own self-regard, and to the standing which it has ever endeavored to sustain among all honorable organizations of its kind, to protect itself against these aspersions, to discountenance and condemn, in the most empahtic manner, the indecent and disgusting practices above mentioned; therefore be it

*Resolved*, That the said Ignatius Langer is no longer worthy of fellowship with us, having forfeited all claims thereto; that hereafter we individually and collectively will hold no futher professional intercourse with him, and that he be and is hereby formally and finally expelled from the membership of this Society."

By the above it will be seen that the expulsion was based upon violations of the Code of Ethics, as well as improper conduct in a case in practice; and yet the editors of the *Medical Press* say, that the paper of Dr. L. is "a thorough refutation of the charges," when the paper contains only an attempt to refute one part of them, and that by making a false issue! How thorough the refutation is, this communication will show.

First, let the profession understand that the Scott County Medical Society has never denied the possibility or propriety of turning by external manipulation *at the time of labor.* Whatever our opinions, as individuals, may be on this subject, the Society has never entertained its consideration, nor has any one ever been denounced for such practice. But it has condemned, and still condemns the practice of requiring females to submit to examinations of their persons, during the period of gestation, for the purpose of making attempts to correct supposed malpositions of the fœtus *previous to the commencement of labor.*—When the expelled member brings forward authorities in support of the practice of turning by external manipulation after labor has commenced, and has the characteristic mendacity to affirm that we discredit such authors, and convey the impression that he was expelled for such practice he is manifestly endeavoring to make a false issue in order to obtain the sympathy of those who can thus be deceived. It is important, therefore, that the profession should understand this distinctly. We will show that the Society had good cause to take action upon the question, independently of all others, and that we are sustained in our decision by the best living authorities of our country.

The value of the paper, *signed* by Mr. and Mrs. Whisler, will be appreciated by comparing it with the following communication from Dr. Gregg, of Rock Island, a veteran member of the profession in our sister State, himself of foreign birth and education, and inferior to none, in every accomplishment becoming a physician and a gentleman.

STATEMENT OF P. GREGG, M. D., OF ROCK ISLAND.

"*Audi alteram partem.*"—Such is the heading of an article, to which my attention has been recently called, written by Dr. Ignatius Langer, of Davenport, Iowa, and published in the New York *Medical Press* of January 28th. Appended to the article is an apparently imposing array of authorities on the subject of external manipulations for the correction of fœtal malpositions, and also a certification from a lady and gentleman, endorsing the gentlemanly conduct and scientific attainments of Dr. L., in a particular case.

The propriety of the above classical caption, on the part of Dr. L., is well enough, as he had heard, and was conversant with the other side of the question. But the *ex parte* endorsement of his article, by the editors of the New York *Medical Press*, is certainly open to very grave objections, as they had before them but the *unum partem* on which to base an opinion as to the merits of the case.

Time does not permit, nor does inclination prompt me to enter upon a discussion of manipulations in general, nor to involve myself in a controversy on this particular item, among many, of the charges which led to the expulsion of Dr. Langer from the Scott County Medical Society. But inasmuch as my disapproval of, or dissent from the practice pursued in that case, and expressed to a member of the Society, originated the charge referred to, justice to myself, and to the Society require from me a full statement of the case, with such comments as may be necessary for its elucidation.

In the month of April, of the past year, I was waited on by Mr. W——r, who very earnestly requested me to visit his lady, in Davenport, in consultation with Dr. Langer. In answer to my inquiry, as to what the trouble was, he replied, (I give his homely, but expressive language,) "My wife is about being put to bed, and the Doctor has a sore finger and cannot operate." On arriving in Davenport, I found the Doctor in his office, and proceeded with him to the patient's residence; the Doctor carrying his arm in a sling, one finger immersed in a tin-cup of iced water, and which, he informed me had been his condition for several days, owing to a severe contusion of the finger.

On arriving at the house, I was ushed into a front parlor, through the open, postern door of which, I saw the patient coming from her kitchen. The Doctor met her in an intermediate room, where he had the lady placed on a bed, and manipulated (with one hand, it will be observed) for some four or five minutes, when he came out and invited me to make an examination, saying at the same time, he thought he had rectified the difficulty.

I must confess to no small surprise, (agreeable of course,) on finding that there was no necessity for my aid, or interference in what I had naturally supposed to be a case of labor. No little out-stretched arm silently, but forcibly pleading for the exercise of obstetrical science in its behalf—no little innocent, elbowing or shouldering his or her way into worldly life! No chance there for "a striking effect," (*vide* Dr. Noeggerath—New York *Journal of Medicine.*) But before me was a strong, healthy, well-formed, and quite fleshy lady, *without a single premonition of labor*, not a hair's breadth of dilatation of the os-uteri; *nothing, in fact, justifying the presence of an old woman, much less of counsel from the other side of the river.*

After expressing my opinion to the lady and her alarmed husband, in which the Doctor coincided, that *all was right*, I left, the Doctor accompanying me. There was as little necessity for his remaining, as for a consultation.

On the day of the Doctor's arraignment before the Society, I was present, and stated the case substantially as here related, inviting Dr. L., if in anything I misrepresented, to correct me. He admitted the truthfulness of my statement, but contended that he did not mean to be understood as correcting the malposition during the short time of my visit, but that he had been gradually accomplishing it, little by little, for a number of days, and on the occasion of my visit he had completed the somersault!

If I may be permitted the use of a slang phraise, intending thereby no disrespect for the Doctor, I would say, this change of position was from "the frying-pan into the fire."

Is this to be the future practice, sanctioned by the New York *Medical Press?* Is a man to be sustained by the profession, when he asserts that he can and did, from hour to hour, and day to day, hitch the fœtus, as it were, on to some imaginary hook, or shelving projection in the uterus, until his leisure permits him to take another hitch, and so on, and this, too, when a woman is moving about, attending to her domestic affairs!

I confess to the obscurity of my locality, and my name in the profession; but I claim that twenty-four years of active practice, and attendance on, at least, twelve or fourteen hundred cases of obstetrics, entitle me to a modest protest, if nothing more, against such practice as developed in this case.

The certificate of Mr. and Mrs. W——r can have no force as a defensive item for the Doctor. Not that I would discredit their statement, on the contrary, I would defend their veracity if assailed; but the "feeling different on this occasion from preceding occasions, and something must be wrong, Doctor," is the old story, and with which every practitioner is conversant; and those "false pains" and "unusual feelings" *open up to the unscrupulous quack the most extensive field of imposition.* The credulity of women in a pregnant state, and their confidence in their medical attendant are unbounded; *and the man who basely takes advantage of that confidence either to produce "a striking effect," or to promote his pecuniary interests should not be acknowledged by the profession nor sustained by the public.*

Signed,

*Rock Island, Febuary* 10, 1860. P. GREGG.

The above statement of Dr. Gregg conveys to the profession a graphic and truthful account of the origin of this controversy. So evident was the unblushing quackery of the whole proceeding, that he was arraigned before the Society by Drs. Barrows and Witherwax, two venerable members of the profession, who had years previously retired from practice, but still, with a highly commendable spirit, retain their connection with the medical organizations of the country, and participate in their proceedings. The former, Dr. E. S. Barrows, is now President of the State Medical Society of Iowa. Dr. L. acknowledged the practice of which he was charged, and affirmed that he would always require a pregnant female to submit to any examination he thought proper to make at any time during her gestation; and, further, that he would not prescribe to such a person unless permitted to do so. He acknowledged, also, as Dr. Gregg testifies, that he repeated his visits, and manipulations, and examinations from day to day, during which time he was slowly progressing in the accomplishment of his purpose, with this very accommodating fœtus, up to the time when Dr. G. was called to the case, when, *keeping Dr. G. out of sight*, he suddenly gives it the last finishing touch of his magic hand, and "all was right;" and the lady resumed her occupations in attending to her household duties, happy, no doubt, in the promise of a safe delivery when her full time should arrive! He furthermore made no denial of the charge that he had, during this period, given the matter a wide notoriety, by relating the character of his visits and their object, to people on the streets, and elsewhere, in violation of all propriety, and with an object too patent to admit of a doubt. The Society then condemned the practice of which he was guilty, and suspended him from membership by an *unanimous vote.* The charges of general unprofessional conduct and violations of the Code of Ethics, were referred to a committee, with instructions to investigate the facts, and report at the next quarterly meeting. The investigation was made during the following three months, and a report submitted, in which his guilt was admitted, but a milder punishment than expulsion recommended, *so far as the charges submitted to their investigation were concerned.* All the testimony was presented in writing and read in full before the Society, and also the defence of the party accused, which latter paper contained as its basis of argument, a series of deliberate and gross falsehoods, of which we have positive proof in the records of the Society. After this, letters were read from a dozen or more Professors of Obstetrics, and others, in different parts of our country, from some of which are taken the extracts which are given below.

In accordance with the sentiments expressed in these letters, and in a firm conviction of acting in strict reference to the requirements of duty and justice, the Society decided that the charges of Drs. Barrows and Witherwax were sustained; and expelled him from membership, for reasons expressed in the preamble, against one negative vote. This vote was given by the chairman of the committee, who accompanied it with this statement, to go upon the record, that he voted in the negative to be consistent with his action as chairman of the committee, which had recommended a milder punishment, but he coincided in sentiment with the authors of the letters which had been read, and acquiesced in the justice of the verdict of the Society. The lateness of the hour had compelled many of the members to be absent, among whom was Dr. Witherwax, one of the two original accusers, (Dr. L. falsely states that both were present.) As Dr. L. has tried to make it appear that he was tried and condemned by a faction, it becomes necessary to mention the above particulars, and to state further, that on this question the Society is a *unit* in sentiment. It is composed of members who reside in Davenport, Le Claire, Princeton, and Blue-Grass, and in various parts of the country within the county, and those who reside without the city of Davenport were prevented from adding their names to the affirmative, only by the lateness of the hour before which they had been obliged to leave to return to their distant homes.

The following extracts are from the letters above mentioned, which were written in reply to certain questions addressed to them in behalf of the Society, and which letters now constitute a part of its records.

The extracts given below are in reply to the question, "What would be your opinion of the conduct of a man who, professing to be able to detect and rectify malpositions of the fœtus in utero before labor, attempts to do so by repeated manipulations, and proclaims to his friends and the public what he is doing?"

We will first give the answer of Chandler R. Gilman, M. D., Professor of Obstetrics in the College of Physicians and Surgeons of New York:

"I say unhesitatingly that the man is a fool and a knave both, and as such utterly unworthy to belong to any association of honorable men. On this subject, it seems to me that there ought not, and cannot be any difference of opinion among scientific physicians and honorable men."

The next is from H. Miller, M. D., Professor of Obstetrics, &c., in the Louisville Medical College, President of the American Medical Association, and Author of a standard work on Obstetrics:

"There can be but one answer to the question by all right-thinking and right-feeling practitioners, namely, that he is an *arrant, unblushing and grossly indelicate charlatan.*"

And he further writes:

"As to the practice of always examining or desiring to examine ladies previous to the commencement of labor, to ascertain whether or not the position of the fœtus is right, I agree with you that such conduct is '*highly indecent and unwarrantable,*' and I may add, that it ought not, and I would fain hope will not be tolerated by '*decent*' females in any part of our country."

P. A. Jewett, M. D., Professor of Obstetrics in the Medical Department of Yale College, New Haven, writes:

"It is *quackery;* and no respectable physician, who values the honor of the profession, would engage in such a course of imposition."

James P. White, M. D., Professor of Obstetrics in the Buffalo Medical College, writes:

"My opinion is, that a man who so professes and conducts himself, is a *quack.*"

Truman Knooles, M. D., Professor of Obstetrics in the Iowa Medical College, writes:

"I should consider such a man a knave, making a base attempt to deceive the public for the purpose of gain; and, further, if I could be made to believe that such an individual *reposed any confidence in his pretensions,* I should think him *insane* or a *fool,* and a subject for the contempt or pity of all honorable men, just as ignorance, insanity, or rascality dictated the course."

Wm. H. Byford, M. D., Professor of Obstetrics in the Lind University of Chicago, writes:

"I unhesitatingly say, that it is *contemptibly unprofessional.* The man who thus demeans himself forfeits the fellowship of honorable medical men, should be anathematized by all professional organizations, and spurned from decent society."

And again he says:

"I can hardly find words strong enough in which to condemn the practice of always asking to examine ladies previous to labor, to see if the child is in the right position. It is certainly *disgustingly indecent*, and I can see no motive but the morbid lustful desire of lascivious libertinism, thus to handle the persons of lady patients."

Prof. Valentine Mott, M. D., L. L. D., writes:

" Such conduct could only be pursued by a *charlatan*. If by a regular practitioner, he would deserve the contempt and reprobation of the profession."

The following is from A. T. Woodward, M. D., Professor of Obstetrics in the Castleton Medical College, Vermont:

" Furthermore, I think any person living in the hope of daring to ask forgiveness for unremitted sin, if compelled by a God-forsaken propensity to practice dishonesty in the healing art, would, unless lust were engraven eternally on his soul, seek some less revolting, a more pardonable way, than that of fingering the credulous and unsuspecting pregnant female, with promises of a safe labor to both mother and child for the sacrifice."

We might go on and quote from Professor Gilbert and Prof. Hodge, of Philadelphia, and numerous others, in condemnation of such conduct and practice. But space will not permit, and why need we mention any more? Are not these sufficient for our justification? Upon these men does such a person as this Dr. Langer dare to cast the imputation of " gross and unpardonable ignorance " and " a criminal neglect of duties !" in the same spirit, and with as much regard for truth as his brother Hungarian, Dr. Eisler, of New York, writes a paper which was read before the Leipsic Obstetrical Society, and published in the Berlin Obstetrical Journal of Oct., 1859, in which luminous paper he says: " In New York, more especially, be it remarked, the science and art of obstetrics has been taught *only during the last three years!* Therefore, the science of obstetrics takes here (in New York) *a very low rank!*" We find with deep regret and astonishment, that even Dr. Noeggerath joins in this denunciation of American practitioners, by charging them with " unjustifiable neglect," and imputing to them most unworthy motives.—(*N. Y. Jour. of Med., Nov.* 1859, *p.* 347.

It will now be apparent that the members of the Scott County Medical Society of Iowa acted deliberately, unitedly and advisedly in this matter; and with the emphatic condemnation of such practice and conduct by the men of highest authority and standing in our country, we felt that it would be a dereliction of duty on our part if we failed to act in accordance with their expressed opinions.

Will the editors of the *New York Medical Press* now venture to say, that we are placed " in an unenviable light before the professional world ?" and will they call this " persecution for opinion's sake ?" Let the profession beware how they credit the statements of one to whom the above expressions of our most eminent teachers apply, and one who has been proven in the society of which he was a member to be utterly regardless of truth. If it were not noticing too much the communication of this man in the N. Y. Med. Press, evidence of this same disposition to deception could there be pointed out. Some have already been alluded to: and we may state that in his lame and labored effort, by aid of Dr. Noeggerath, to bring apparently an array of authority to sustain him, he includes those like Ramsbotham, who emphatically deny the possibility of detecting malpositions before labor. Thus, this author says (p. 262, 4th Am. Ed.) " It is then only after labor has commenced, and when, indeed, it has made some progress, that we can positively detect a transverse presentation;" and yet he names this distinguished author as one of those who support his position ! and more than that, he is named as one of those who have no credit with the members of our society, when he knew he was saying an untruth, and practicing a piece of deception which is in keeping with the character of the practice of which we found him guilty. But the paper, with all its absurdities, misrepresentations and gasconade, is unworthy of further notice, and would not be noticed at all but for the editorial endorsement it has received, accompanied with an unbecoming reflection upon our official action and professional character.

In the March number of the N. Y. Jour. of Med. will appear a paper from the Scott County Medical Society, in reply to Dr. Noeggerath, who has publicly called our attention to his last report, in a manner which betrays the spirit which prompted its publication. To this we invite the attention of the profession of the United States, upon whom Dr. Noeggerath and others, who are so much his inferiors that they should not be named together, have presumed to cast the imputation of " gross and unpardonable ignorance" and " unjustifiable neglect," and what Dr.

N. calls "a very pardonable desire" to practice so as to produce "a striking effect!" Dr. N. may yet learn that American practitioners are not amenable to such a charge, and if such desires appear "pardonable" in his estimation, they will receive no such endorsement by the members of the profession he thus insults. It may, however, be pardonable for them to prefer the sound, practical common sense of English and American writers, to the vagaries of the German *Illuminati.*

And what is the practice which Dr. Noeggerath would have the profession of this country adopt?

It is worthy of note that what one of this class of obstetricians (*Mattei*) considers proper and commendable, another luminary of this order (*Esterle*) regards as "unnecessary and dangerous."

It is also on record that in a letter to a member of the Scott County Medical Society, addressed to him in its behalf, Dr. Noeggerath says, "the very labor pains are *required* to insure the success of the operation," by which of course is implied that it cannot be performed before labor. Soon after this, in the Nov. No., 1859, of the N. Y. Jour. ot Med., he says, in effect, that it may be performed before labor, but it will do no good; and directs us to wait until labor has commenced. Two months later, in the same journal, he teaches that it should be performed before labor, "*at any time during the last three months of pregnancy, as soon as the accoucheur detects a transverse presentation!*" How can Dr. N. expect the profession to keep pace with such wonderful progress? In his last communication he recommends, through Esterle, when ordinary manipulations fail, to resort to "*gentle knocks*" upon the ends of the fœtus alternately, and "*concussions*" upon the head "in quick succession." Is this the climax of the practice, or must we look for something still more marvellous in the next Report? All this is to be done "at any time during the last three months of pregnancy," (last November it was to be done only "at the time of the beginning of labor." If the rectification is made in the seventh month, how frequently must it be repeated during the eighth and ninth? and how frequently must the search be made for mal-positions? Evidently, according to this doctrine, every unborn child must have a doctor to *pilot its direction* during its last three months of fœtal life, and all pregnant females must be examined repeatedly during the last three months of gestation, as invariably, and as much a matter of course, as we now feel the pulse of an invalid!

Such is the character of the practice which has been condemned by our society, and such the consistency of its advocates! We are informed by Prof. D. Humphreys Storer (on what authority he does not say,) that, "In the Hospitals of Vienna, women are constantly presenting themselves for examination to the physicians to have their "presentations" determined; and the physicians affirm (?) that in the great majority of cases they can and do determine this *previous to the seventh month! Cui bono?* one may well ask. Such needless and worthless experiments (if such are made) may be tolerated by those females who are totally lost to all sense of decency and shame, in a city where the standard of morality is so low that nearly or quite one-half of all the births are illegitimate; but it is presumptuous, to say the least, for such advocates of obstetrical fumbling to ask the people of this country to adopt a practice so repugnant to their ideas of morality. Advocating a practice of this character, and disagreeing among themselves as to how much of their operations are "unnecessary and dangerous," they presume to lecture the profession of this country for not having adopted this *unnecessary and dangerous, and highly immoral and disgusting practice;* and dare to utter the charge of "gross and unpardonable ignorance" and criminal and unjustifiable neglect of duty. And such language as this has received the approving commendation of the editors of the N. Y. Medical Press, and a Medical Society is denounced for its condemnation of such a practice. Are they *Americans* who thus submit so graciously to these insulting imputations? But it matters not. We are members of one common brotherhood in our noble calling, and the interests of humanity as well as the honor of the whole profession demand a careful scrutiny of the conduct of those who would dishonor its character and degrade its dignity by the tricks of a charlatan, though practiced under the imposing garb of scientific pretensions, and in the name of medical progress. The members of the Scott County Medical Society of Iowa stand before the profession of this country firmly committed to the denunciation of this repulsive innovation, which they regard as mockery of science and a caricature on medical progress.

It is our object by this communication to place in its true light the exact nature and origin of this controversy, which has been so grossly misrepresented; and to show that we have been sustained by the best living authorities of our country.

For a review of the paper of Dr. Noeggerath which he recommended to our perusal, and a full exposition of the absurdity of the practice and its unfounded claims to scientific pretensions, the attention of the profession is again invited to an official communication which has been sent for publication to the New York Journal of Medicine.

J. M. ADLER, M. D.,

E. J. FOUNTAIN, M. D.,

C. C. PARRY, M. D.,

J. W. H. BAKER, M. D.,

THOS. J. SAUNDERS, M. D.

} Committee.

*Davenport, Iowa, Feb.* 10, 1860.

[Philadelphia Medical and Surgical Reporter.

